# The upregulated *LsKN1* gene transforms pinnately to palmately lobed leaves through auxin, gibberellin, and leaf dorsiventrality pathways in lettuce

**DOI:** 10.1111/pbi.13861

**Published:** 2022-07-02

**Authors:** Menglu Wang, Dean Lavelle, Changchun Yu, Weiyi Zhang, Jiongjiong Chen, Xin Wang, Richard W Michelmore, Hanhui Kuang

**Affiliations:** ^1^ Key Laboratory of Horticultural Plant Biology, Ministry of Education College of Horticulture and Forestry Sciences Huazhong Agricultural University Wuhan China; ^2^ Genome Center and Department of Plant Sciences University of California Davis California USA

**Keywords:** palmately lobed leaves, pinnately lobed leaves, *KNOX1*, transposon, phytohormones

## Abstract

Leaf shape represents a vital agronomic trait for leafy vegetables such as lettuce. Some lettuce cultivars produce lobed leaves, varying from pinnately to palmately lobed, but the genetic mechanisms remain unclear. In this study, we cloned one major quantitative trait locus (QTL) controlling palmately lobed leaves. The candidate gene, *LsKN1*, encodes a homeobox transcription factor, and has been shown previously to be critical for the development of leafy heads in lettuce. The *LsKN1* allele that is upregulated by the insertion of a transposon promotes the development of palmately lobed leaves. We demonstrated that LsKN1 upregulated *LsCUC2* and *LsCUC3* through different mechanisms, and their upregulation was critical for the development of palmately lobed leaves. LsKN1 binds the promoter of *LsPID* to promote auxin biosynthesis, which positively contributes to the development of palmately lobed leaves. In contrast, LsKN1 suppresses GA biosynthesis to promote palmately lobed leaves. LsKN1 also binds to the promoter of *LsAS1*, a dorsiventrality gene, to downregulate its expression. Overexpression of the *LsAS1* gene compromised the effects of the *LsKN1* gene changing palmately to pinnately lobed leaves. Our study illustrated that the upregulated *LsKN1* gene led to palmately lobed leaves in lettuce by integrating several downstream pathways, including auxin, gibberellin, and leaf dorsiventrality pathways.

## Graphical abstract

Cultivated lettuce (*Lactuca sativa*), domesticated from prickly lettuce (*L. serriola*), is one of the most important green leafy vegetables worldwide. Lettuce is also a model species for hydroponics, and an ideal plant to be engineered to produce oral vaccines or valuable pharmaceuticals. Leaf shape is an important trait for plant species, and leaf shape per se is an agronomic trait for leafy vegetables such as lettuce. Lobed leaves have been studied in several species. However, the mechanism underlying the variation between pinnately and palmately lobed leaves remain unclear. For the first time, we dissected the genetic mechanisms underlying palmately lobed leaves in plants, providing novel insights on leaf development. The palmately lobed leaves contribute to the sensory quality of lettuce, and our results will facilitate the breeding of lettuce cultivars with ideal leaf shapes.

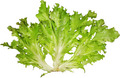

## Introduction

Plant leaf is the primary organ to harvest light energy through photosynthesis (Tsukaya, 2013). Though the function of leaves is conserved, their shapes may vary dramatically among different plant species (Chitwood and Sinha, 2016; Tsukaya, 2018). The underpin mechanisms for the high divergence of leaf shapes are not well understood (Drost *et al*., 2015). Leaf shape may have evolved to adapt to natural habitats (Nicotra *et al*., 2011). However, closely related plant species with similar distribution regions or niches can vary considerably in leaf shapes (Byrne, 2012). Furthermore, leaf shape may vary among individuals within a natural population of the same wild species (Hickey, 1973). Leaf shapes of some cultivated species have experienced considerable modifications during domestication to increase yield or fit agricultural practices (Rowland *et al*., 2020). For leafy vegetables, leaf shape *per se* is the target of artificial selection (Sedivy *et al*., 2017).

The prominent polymorphisms of leaf shape are single leaf versus compound leaves, and non‐lobed leaves versus lobed leaves. Fossil evidence suggested that the ancestors of angiosperm were unifoliate, and, therefore, compound leaves had evolved from single leaves and developed convergently in different plant lineages (Ledford, 2018). Compared with single leaves, compound leaves possess many benefits, such as better gas exchange and less tissue damage from herbivores (Higuchi and Kawakita, 2019). It was hypothesized that compound leaves were more developmentally elastic and flexible than single leaves, allowing a wide range of mutations to produce new phenotypic manifestations (Sisó *et al*., 2001). Though lobed leaves were anatomically and developmentally different from compound leaves, the regulation of the development of lobed leaves in some plant species resembled that of compound leaves (Chang *et al*., 2019). Lobed leaves contained higher plasticity of spatial extension and responded more quickly to compete for limited light sources than non‐lobed leaves (Baker‐Brosh and Peet, 1997). Plants with lobed leaves acquired better adaptions to low temperatures in high latitudes or cold regions than those with non‐lobed leaves (Sedivy *et al*., 2017). The complexity of lobed leaves had a negative correlation with leaf hydraulic resistance, and lobed leaves were hypothesized to help plants achieve water balance under dry atmospheric conditions (Nicotra *et al*., 2011).

The molecular mechanisms for the development of lobed or compound leaves have been studied elusively in model species such as tomato (*Lycopersicon esculentum*), *Cardamine hirsute*, *Medicago truncatula*, and *Lotus japonicus* (Champagne *et al*., 2007). Lobed leaves have auxin maxima at the tip of leaf lobes, accompanied with high expression level of *CUP‐SHAPED COTYLEDON* (*CUC*) genes in the sinuses. Overexpression of CUC1 and its homologs CUC2 and CUC3 promote leaflet separation and leaflet formation, leading to the development of leaf lobes (Aida *et al*., 1997; Blein *et al*., 2008; Takada *et al*., 2001; Vroemen *et al*., 2003). Reduced expression of NAM/CUC boundary genes suppresses all marginal outgrowths and consequently reduces the number of leaf lobes (Vroemen *et al*., 2003).

The leaf lobe pattern was orchestrated by several pathways, and one of them involved the KNOXI homologs, a family of homeobox transcription factors (Vollbrecht *et al*., 1991). Members of the KNOX1 family were mainly expressed in shoot meristems and subtending stems to maintain meristematic activity. The KNOXI family participated in the initiation of lateral organs (Hake *et al*., 2004). KNOX1 mRNA transports between cells through ribosomal RNA‐processing protein 44A, which was critical to regulate the stem cell‐dependent processes in plants (Kitagawa *et al*., 2022). Up‐regulation of the *KNOX1* genes after the formation of leaf primordia is unique for plants with lobed or compound leaves (Efroni *et al*., 2010; Hareven *et al*., 1996; Janssen *et al*., 1998; Piazza *et al*., 2010; Shani *et al*., 2010; Veit, 2009). Increased expression of a *KNOXI* homolog in tomato led to super‐compound leaves with thousands of lobed leaflets (Janssen *et al*., 1998). The *KNOX1* gene in tomato is negatively regulated by a BEL‐like homeodomain protein BIPINNATA (BIP), and the mutation of the *bip* gene boosted the complexity of leaf morphology in tomato (Nakayama *et al*., 2021). *Arabidopsis* contained four KNOXI homologs including SHOOTMERISTEMLESS (STM), KNAT1, KNAT2, and KNAT6 (Hake *et al*., 2004). Overexpression of *KNAT1* in Arabidopsis transformed entire leaves into lobed leaves by repressing the *AS1* and *AS2* genes (Chuck *et al*., 1996; Ori *et al*., 2000). The expressions of the *KNOXI* genes are regulated by several other transcription factors besides *AS1* and *AS2*. For example, BLADE ON PETIOLE1 (BOP1) and BOP2, members of the BTB ankyrin family repress the expression of the *KNOX1* family and play critical roles in maintaining a border between meristem organ compartments (Khan *et al*., 2014).

Besides the *KNOXI* family, several other families were also shown to regulate the development of lobed leaves or compound leaves. The polymorphism of lobed leaves and non‐lobed leaves in cotton, rapeseed, and watermelon was genetically controlled by the polymorphic *LMI1* homologs, which also encode homeobox transcription factors (Andres *et al*., 2017; Sicard *et al*., 2014; Vlad *et al*., 2014; Wei *et al*., 2017). The *UNIFOLIATA* (*UNI*) in pea and *SINGLE LEAFLET1* (*SGL1*) in Medicago, orthologs of the Arabidopsis floral meristem identity protein LEAFY (LFY), played critical roles in leaf shapes (Hofer *et al*., 1997). Pea *uni* mutant and Medicago *sgl* mutant reduced the complexity of compound leaves (Gourlay *et al*., 2000; Wang *et al*., 2008). In addition, PALMATE‐LIKE PENTAFOLIATA1 (PALM1), a zinc finger protein, controlled the development of the trifoliate leaves in Medicago through negatively regulating *SGL1* in lateral leaflet regions (Chen *et al*., 2010). PINNATE‐LIKE PENTAFOLIATA1 (PINNA1), a BEL1‐like homeodomain protein, attenuated the expression of *SGL1* in the terminal leaflet regions (He *et al*., 2020). *SlLAM1*, the ortholog of *WOX1* in tomato, facilitated secondary leaflet initiation and maintained the morphology of compound leaves (Wang *et al*., 2021). Moreover, the SMOOTH LEAF MARGIN1 (SLM1) protein in *M. truncatula*, which is an auxin efflux carrier protein and is the ortholog of PIN‐FORMED1 (PIN1) from *A. thaliana*, regulates the complexity of leaves (Zhou *et al*., 2011). Loss of function in *MtGA3ox1*, an enzyme for GA biosynthesis, promotes serration on the blade margin and increases leaf complexity. The above data suggested the antagonistic role of phytohormones GA and auxin in the regulation of lobed leaves and compound leaves, with auxin increasing, and GA decreasing leaf complexity (Bar and Ori, 2015).

Cultivated lettuce (*Lactuca sativa*), domesticated from prickly lettuce (*L. serriola*), is one of the most important green leafy vegetables worldwide (Zhang *et al*., 2017). Lettuce is also a model species for hydroponics, and an ideal plant to be engineered to produce oral vaccines or valuable pharmaceuticals (Daniell *et al*., 2001; Kanamoto *et al*., 2006; Lal *et al*., 2007; Power *et al*., 2021). Both cultivated and wild lettuce have the polymorphism of lobed and non‐lobed leaves. The polymorphism of lobed leaves and non‐lobed leaves in cultivated lettuce was inherited from its progenitor *L. serriola*, and the causal gene is located on Chromosome 3 (Wei *et al*., 2021). Previous studies focused on pinnately lobed leaves in lettuce, but it is a daunting challenge to uncover the genetic and molecular mechanisms underlying the development of palmately lobed leaves in lettuce.

In this study, we conducted a bulked segregant analysis (BSA) combined with RNA‐seq (BSR) to dissect the genetics underlying the complexity of leaf lobes in lettuce. We fine mapped and cloned a major QTL controlling lobe complexity of lettuce leaves. We verified the candidate gene with a complementation test and investigated its molecular mechanism in detail. Our results opened the door to the molecular regulation of lobed leaves, and are useful in breeding programs to develop lettuce cultivars with ideal leaf shapes.

## Results

### Genetic analysis of the complexity of leaf lobe in lettuce

**Figure 1:**
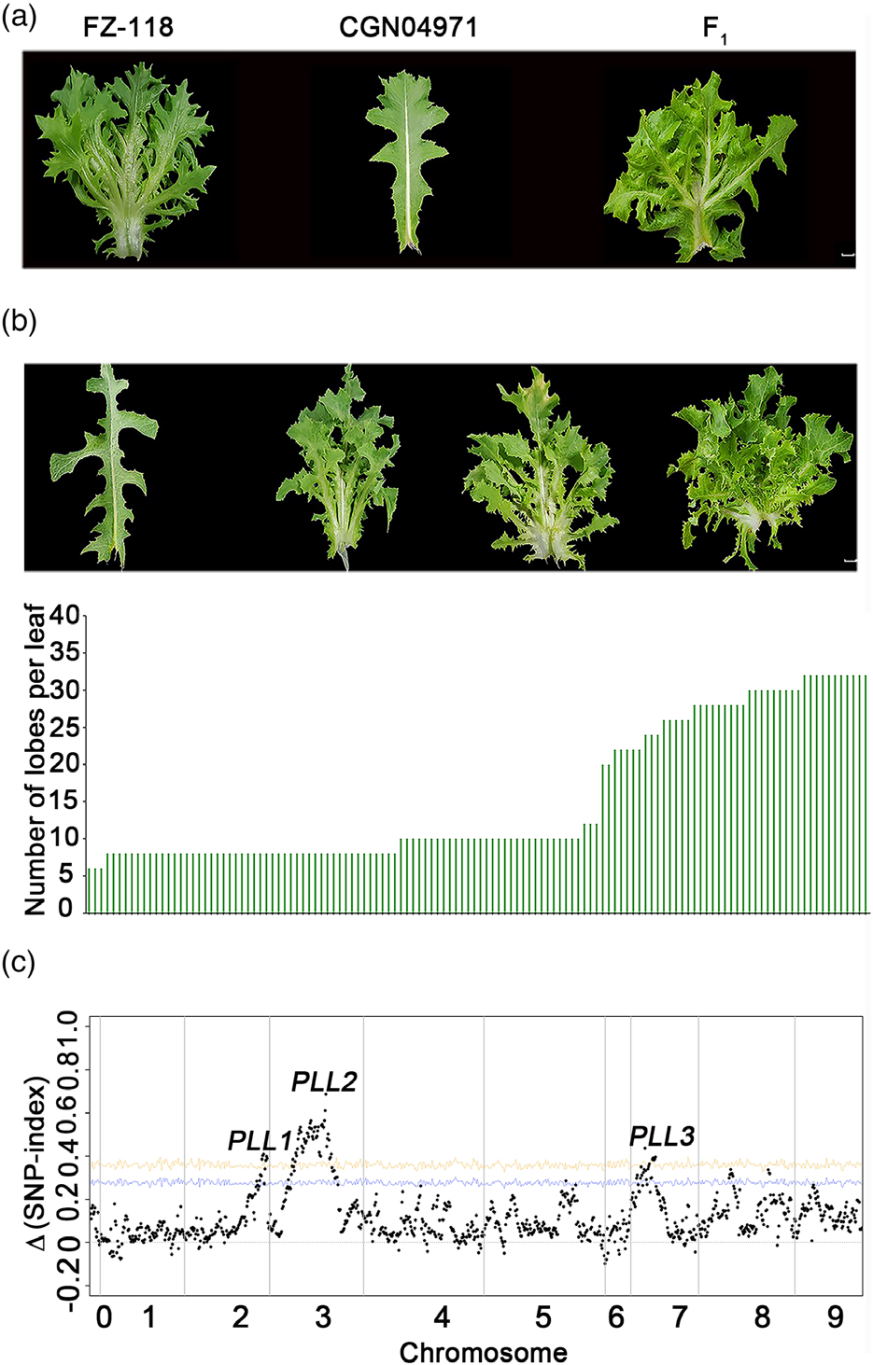
Complexity of leaf lobes. (a) Leaves of parents FZ‐118, CGN04971, and their F_1_ hybrid. (b) Continuous distribution of lobe complexity in the F_2_ population. The bottom figure shows the distribution of lobe complexity (number of lobes per leaf). Bars = 2 cm. (c) Plot of ∆ (SNP‐index) between the extremely palmately lobed pool and pinnately lobed pool constructed from the F_2_ population. Three loci associated with the complexity of leaf lobe are detected in the F_2_ population.

To investigate the genetic mechanism underlying palmately lobed leaves in lettuce, we crossed an inbred line (FZ‐118) of palmately lobed leaves with a wild lettuce accession (*L. serriola*, CGN04971) of pinnately lobed leaves (Figure 1a). The F_1_ population had palmately lobed leaves similar to those of the inbred line FZ‐118, suggesting that the gene(s) controlling palmately lobed leaves is dominant. The F_1_ individuals were self‐pollinated to generate an F_2_ segregating population. Individuals from the F_2_ population showed a continuous distribution of lobe complexity, ranging from pinnately lobed leaves to palmately lobed leaves, clarifying the lobe complexity as a quantitative trait in lettuce (Figure 1b).

We performed BSR to dissect the genetics underlying the lobe complexity in the F_2_ population. The differences of allele frequencies, ∆(SNP‐index), between an extremely lobed pool and a pinnately lobed pool, were plotted along the nine chromosomes of lettuce. The plot figure demonstrated that three major loci contributed to the complexity of leaf lobes in the F_2_ segregating population, located on chromosomes 2, 3, and 7, respectively (Figure 1c).

To verify the potential loci controlling the lobe complexity, we designed Cleaved Amplified Polymorphic Sequence (CAPS) markers at the three potential loci, and screened the F_2_ population. Analysis of Variance (ANOVA) illustrated that the three loci were significantly associated with lobe complexity (*P* < 0.001). We defined the loci on chromosomes 2, 3, and 7 as *Palmately Lobed Leaf 1, 2*, and *3*, (*PLL1*‐*3*), respectively. ANOVA of the F_2_ population suggested that the *PLL1*, *PLL2,* and *PLL3* explained 16.5%, 9.7%, and 22.0% of the variance on lobe complexity in the F_2_ population. This study focused on the *PLL3* locus on chromosome 7, which contributes the highest phenotypic variation explained (PVE) of 22.0%.

### The candidate gene for *PLL3* is the *LsKN1*gene

**Figure 2:**
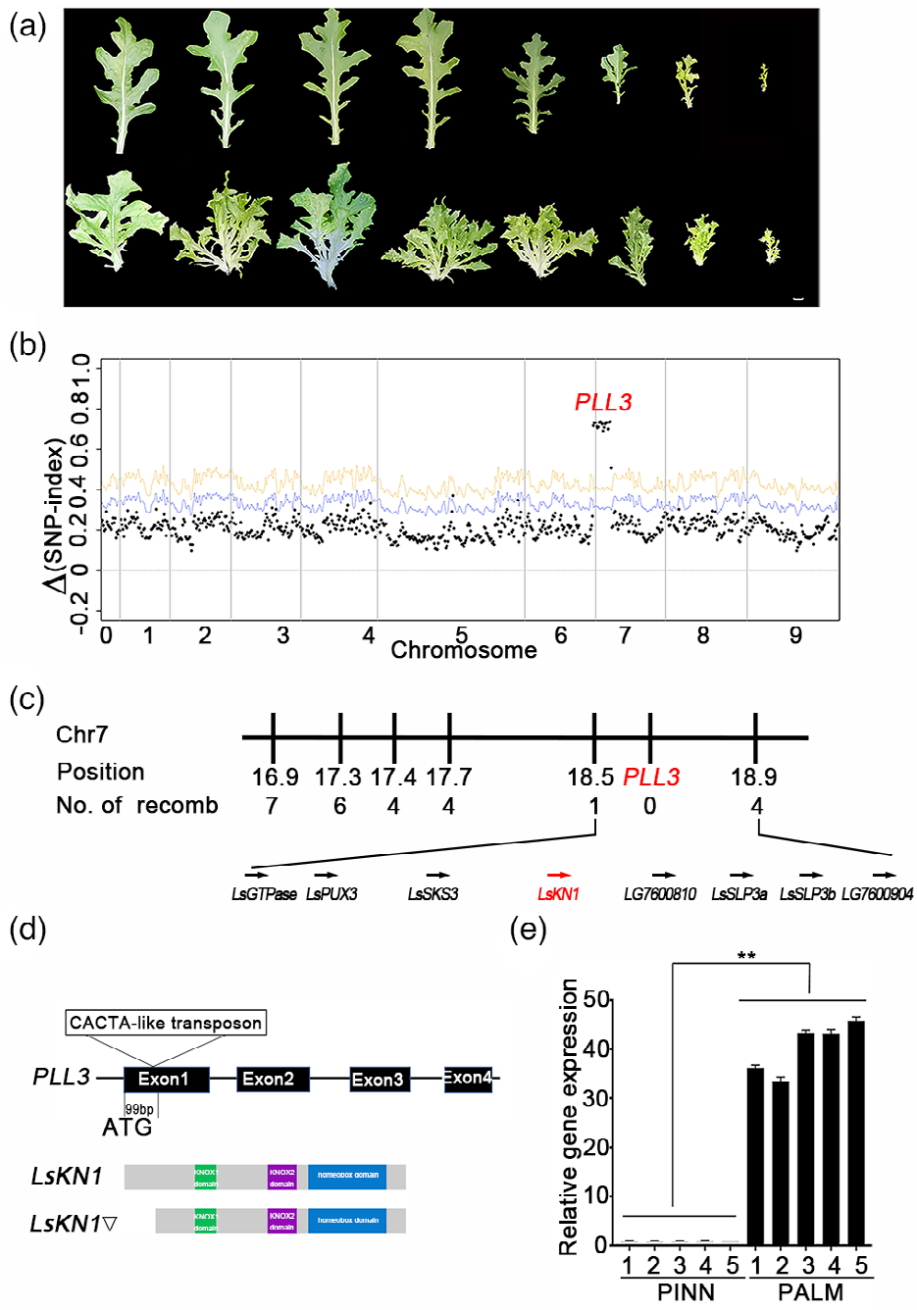
Genetic cloning of the *PLL3* gene. (a) An F_3_ family had individuals with pinnately lobed leaves (top) and palmately lobed leaves (bottom). Bars = 2 cm. (b) Plot of ∆(SNP‐index) between the pinnately lobed pool and palmately lobed pool from an F_3_ family. (c) Linkage map of *PLL3*. Number of recomb refers to the number of recombinants between a marker and the *PLL3* gene among 1004 individuals from the F_3_ family. (d) Schematic representation of *LsKN1*, the candidate gene of *PLL3*. The CACTA‐like transposon is present in the *LsKN1*▽ allele from the parent with palmately lobed leaves. Black boxes represent the exons of the candidate gene. The bottom panel shows the predicted proteins encoded by the two alleles. The mutated protein lost the 79 amino acids at the N‐terminal. (e) The expression of the *LsKN1* gene in the F_3_ family. Five individuals with pinnately lobed leaves and five individuals with palmately lobed leaves were randomly chosen from the F_3_ family. Data represent mean ± SD (n = 3). ** denotes *P* < 0.01.

An individual from the F_2_ population, which was heterozygous at the *PLL3* locus but homozygous at the *PLL1* and *PLL2* loci, was self‐pollinated to generate an F_3_ family. Individuals from the F_3_ family produced either palmately lobed or pinnately lobed leaves, with no intermediate phenotypes (Figure 2a). Of the 405 individuals from this F_3_ family, 302 had palmately lobed leaves, and 103 had pinnately lobed leaves, which fits the Mendelian ratio of 3:1 (*χ* ^2^ = 0.98, *P* > 0.05). BSR method was used to investigate the genetics of lobe complexity in the F_3_ family, pointing to a single locus on chromosome 7 (*PLL3*) (Figure 2b). We therefore used this F_3_ family to fine map and clone the *PLL3* gene. We genotyped additional 599 individuals from the F_3_ family using a series of genetic markers in the candidate region, and consequently delimited the *PLL3* gene between markers CAP18.5 and CAP18.9, in an interval of 400 kb on chromosome 7 (Figure 2c).

The candidate region contains eight genes, with *LsKN1* as the only differentially expressed one between the pinnately lobed and the palmately lobed leaves (Table S1). The *LsKN1* gene was reported to be critical for the development of leafy heads in crisphead lettuce (Yu *et al*., 2020). We investigated the polymorphism of the *LsKN1* gene between the two parents. The parent CGN04971 with pinnately lobed leaves had the wild‐type allele. The parent FZ‐118 with the palmately lobed leaves had the mutated allele *LsKN1*▽, with an insertion of 3935 bp CACTA‐like transposon at +99 bp. The above result was identical to the allele indispensable for the development of leafy heads in lettuce (Figure 2d) (Yu *et al*., 2020). The LsKN1▽ protein contained the conserved *KNOX1*, KNOX2, and homeobox domains found in LsKN1 (Figure S1). A marker specific to *LsKN1* co‐segregated with the lobe complexity in the F_3_ family. The expression of the *LsKN1▽* allele in palmately lobed leaves was approximately 40 folds as much as that of the wild‐type allele in pinnately lobed leaves (Figure 2e). Therefore, the insertion of the CACTA transposon did not knock out *LsKN1▽* but upregulated its expression. We hypothesized that the upregulated expression of the *LsKN1* gene transformed pinnately lobed leaves to palmately lobed leaves.

### Verification of the function of *LsKN1* on the complexity of leaf lobe

We then used a complementation test to verify the function of *LsKN1* on palmately lobed leaves. We chose an individual from the F_3_ family with a homozygous *LsKN1* allele producing pinnately lobed leaves for further study. This individual was self‐pollinated to generate a homozygous line (PINN) with pinnately lobed leaves. Similarly, an isogenic line named PALM, with homozygous LsKN1▽ and palmately lobed leaves, was generated for further study.

**Figure 3:**
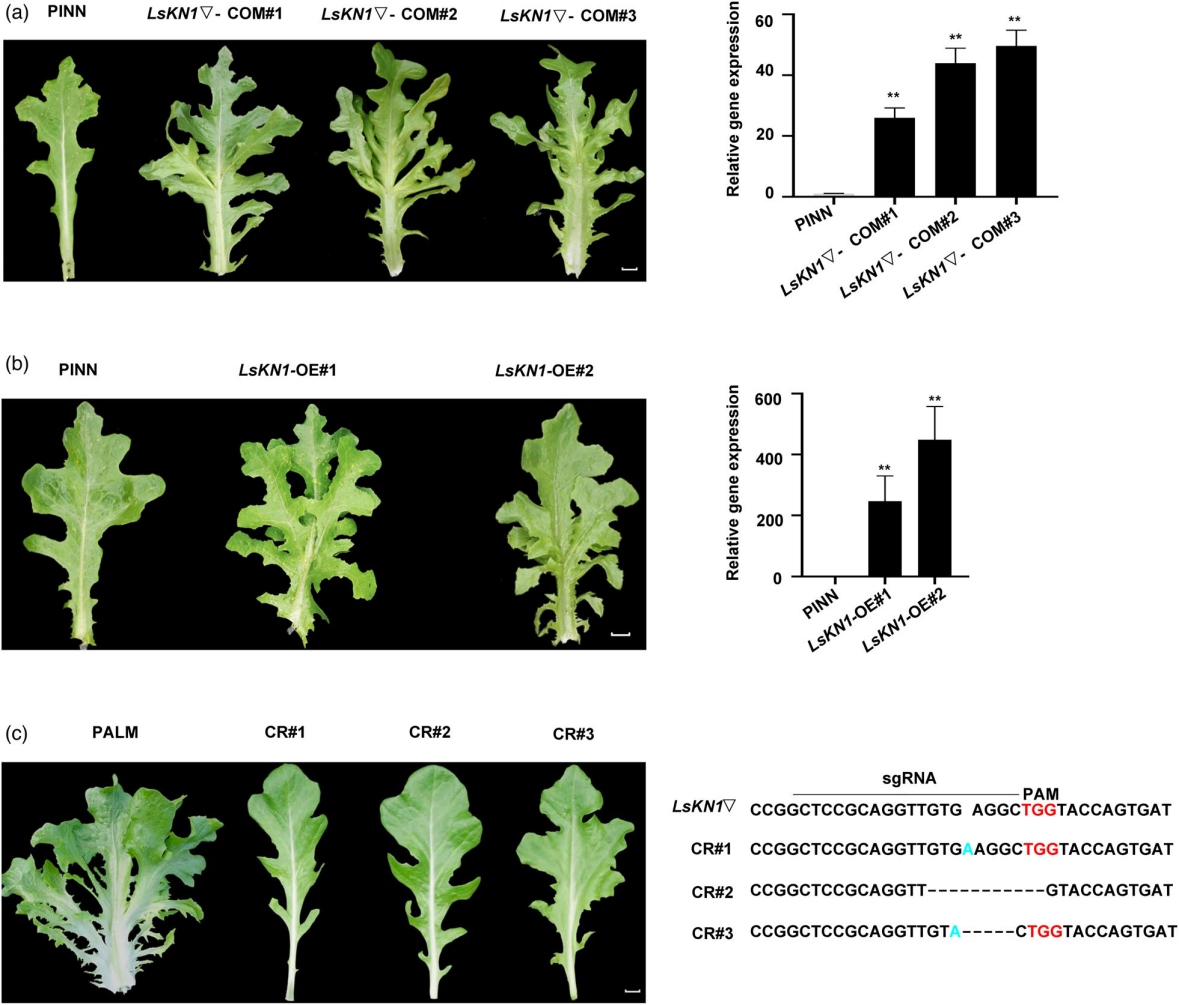
Verification of the function of *LsKN1* on lobe complexity. (a) Complementation test. Transformation of *LsKN1*▽ changed pinnately lobed leaves (PINN) to palmately lobed leaves (COM#1‐3) (left panel). qRT‐PCR shows high expression of *LsKN1* in positive complementation lines (right panel). Data represent mean ± SD (n = 3). ** denotes *P* < 0.01. (b) Overexpression of *LsKN1*. Overexpression of *LsKN1* changed pinnately lobed leaves (PINN) to palmately lobed leaves (OE#1‐2) (left panel). qRT‐PCR shows high expression of *LsKN1* in the two overexpression lines (right panel). Data represent mean ± SD (n = 3). ** denotes *P* < 0.01. (c) Knockout of *LsKN1* using CRISPR/Cas9. Knockout of *LsKN1* changed the palmately lobed leaves (PALM) to pinnately lobed leaves (CR#1‐3) (left panel). Modification of the sequences in the *LsKN1* gene in the knockout plants (right panel). The sgRNA sequences are indicated with a horizontal line. Dash lines refer to deletion, and inserted nucleotides are in blue. PAM sequences are in red. Bars = 2 cm.

We transformed a fragment of 10 220 bps containing the *LsKN1* allele, including the CACTA‐like transposon, into the PINN line. We obtained three independent transformants, and all of them produced palmately lobed leaves (Figure 3a). All three T_1_ populations showed a 3:1 Mendelian segregation ratio (*P* > 0.05), and the palmately lobed leaves co‐segregated with the insert, confirming *LsKN1* as the *PLL3* gene controlling palmately lobed leaves in lettuce. The expression of *LsKN1* showed a deep increase in the transformants, supporting the correlation between its expression level and the leaf lobe complexity (Figure 3a). We further overexpressed the *LsKN1* gene in the PINN line. The two overexpression lines had palmately lobed leaves, in contrast to the pinnately lobed leaves in the wild‐type PINN (Figure 3b).

We then knocked out the *LsKN1* gene in PALM to further confirm its function on lobe complexity. We constructed a recombinant CRISPR/Cas9 vector with sgRNA specific to the coding region of the *LsKN1* gene and transformed it into the PALM line. Three knockout lines were obtained, and all of them had pinnately lobed leaves, in contrast to palmately lobed leaves in PALM (Figure 3c). Our knockout results further verified that the upregulated *LsKN1▽* contributed to the palmately lobed leaves in lettuce.

### A large number of LsKN1 target genes are differentially expressed between palmately and pinnately lobed leaves

We sequenced the RNA extracted from the young leaves of the *LsKN1* knockout mutants with pinnately lobed leaves in the T_1_ generation and its recipients, which have the *LsKN1*▽ allele and palmately lobed leaves. There are 389 differentially expressed genes (DEGs) between the pinnately lobed and palmately lobed individuals (Table S2). Of them, 76 genes are the potential targets of LsKN1 according to the ChIP‐seq results of LsKN1 (Table S3; Figure S2). Some of the 76 target genes were predicted to be associated with leaf development, such as *LsCUC3* (*LG9822694*), *LsHB* (*LG8751215*)*, LsGA20ox1* (*LG9790044*), *LsYAB3* (*LG6576535*), *LsAS1* (*LG4386200*), and *LsCKX3* (*LG5469613*).

Our previous study also identified 581 DEGs between LsKN1▽ mutant and its wild type (Table S4; Yu *et al*., 2020). Note that the 581 DEGs were detected between two genotypes with non‐lobed leaves. A comparison between the 581 DEGs under non‐lobed leaf background with the 389 DEGs under lobed leaf background revealed 303 DEGs specific to the latter (Figure S2). These lobed‐leaf specific 303 DEGs included genes critical for leaf development, such as *LsCUC3* (*LG9822694*), *LsGA20ox1* (*LG9790044*), *LsYAB3* (*LG6576535*), *LsCUC2* (*LG7605596*), *LsPIN5* (*LG1109276*), *LsTCP10* (*LG1159713*), *LsTCP18* (*LG4391812*), and *LsCKX3* (*LG5469613*).

### LsKN1 binds to the promoter of *LsCUC3* and upregulates its expression

**Figure 4:**
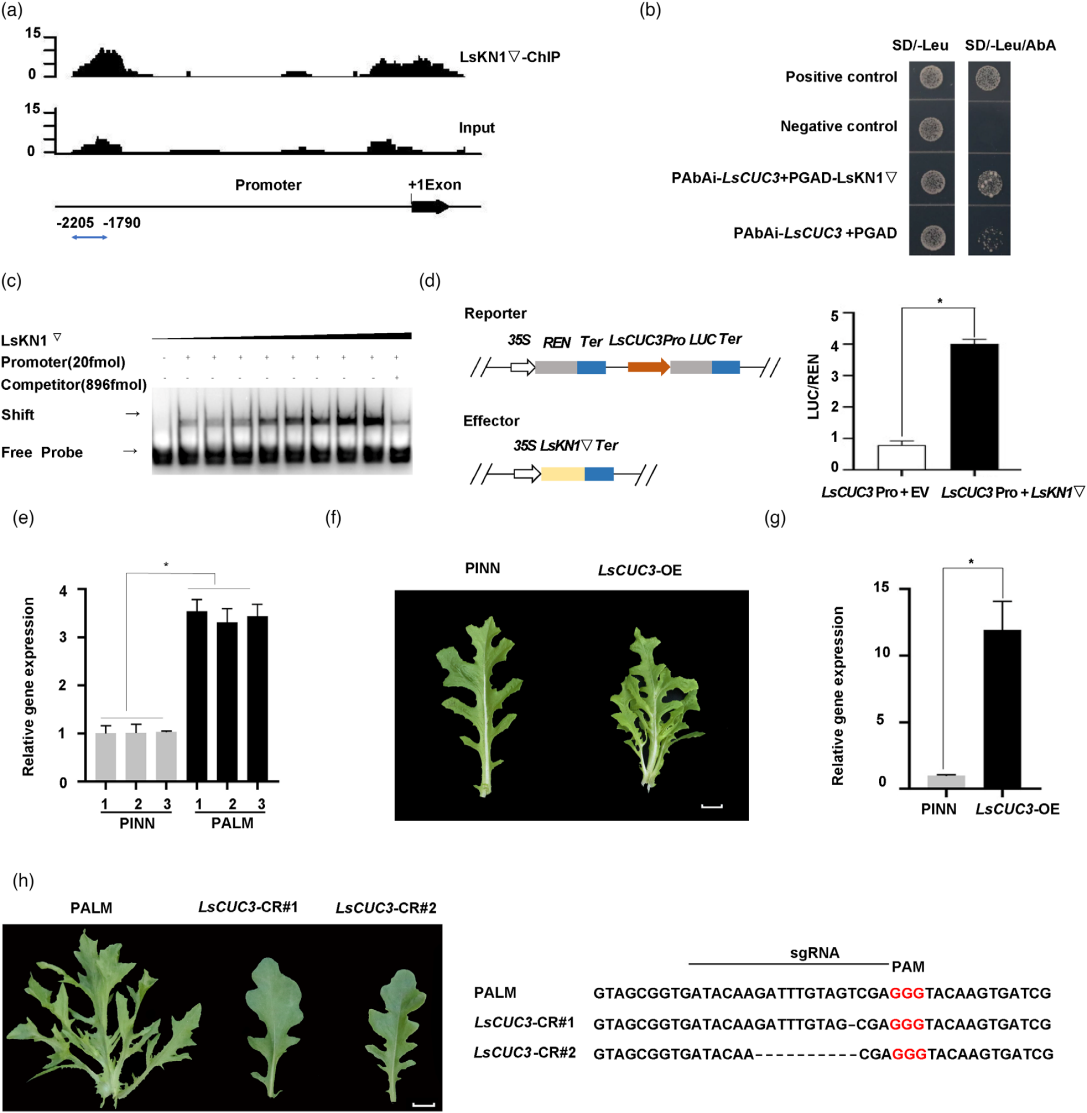
LsKN1 binds to the promoter of *LsCUC3* and upregulates its expression. (a) ChIP‐seq analysis of LsKN1 binding activity in the promoter region of *LsCUC3*. The y‐axis represents the number of reads in the ChIP‐seq. The x‐axis shows the position of the reads in the gene. (b) Y1H assay for LsKN1 and the promoter sequences of *LsCUC3*. Transformants were grown on the SD/‐Leu medium with 500 ng/mL AbA. Positive control, transformants of p53‐AbAi and pGADT7‐p53; negative control, transformants of p53‐AbAi and pGADT7. The region of promoter is shown in (a). (c) EMSA showing the binding of LsKN1 to the promoter of *LsCUC3*. (d) Dual‐luciferase assay showing the effects of LsKN1 on *LsCUC3*. The left panel shows the diagram of reporter and effector vectors used in the dual luciferase assay. The right panel shows the LUC activity when the *LUC* gene driven by the promoter of *LsCUC3* was co‐expressed with an empty vector (EV) or *LsKN1*▽. Data represent mean ± SD (n = 3). * denotes significance level of *P* < 0.05. (e) qRT‐PCR analysis of *LsCUC3* expression in *LsKN1* and *LsKN1*▽ genotypes randomly chosen from the F_3_ family. Data represent mean ± SD (n = 3). * denotes significance level of *P* < 0.05. (f) Overexpression of *LsCUC3* changed pinnately lobed leaves to palmately lobed leaves. (g) qRT‐PCR analysis of the *LsCUC3* gene in overexpression line. RNA was extracted from the sinuses of lobes. Data represent mean ± SD (n = 3). * denotes significance level of *P* < 0.05. (h) Knockout of *LsCUC3* using CRISPR/Cas9. Knockout of *LsCUC3* changed palmately lobed leaves to pinnately lobed leaves (left panel). Sequence modification in the *LsCUC3* gene in the knockout mutants (right panel). The sgRNA sequences are indicated with a horizontal line. Dash lines refer to deletion. PAM sequences are in red. Bars = 2 cm.

Previous studies suggested that the *CUC* gene family was associated with serrations or lobed leaves (Blein *et al*., 2008). The lettuce genome contains three *CUC* homologs. *LsCUC3* represented an ortholog of Arabidopsis *CUC3*, while *LsCUC2a* and *LsCUC2b* were duplicated after divergence of *Asteraceae* and *Brassicaceae* as the ortholog of Arabidopsis *CUC2* (Figure S3). ChIP‐seq of LsKN1 suggested that *LsCUC3* was a target of LsKN1, but *LsCUC2a* and *LsCUC2b* were not (Figure 4a; Table S3). LsKN1 directly bound the promoter region of *LsCUC3* in the yeast one‐hybrid (Y1H) assay (Figure 4b). Further electrophoretic mobility shift assays (EMSAs) demonstrated that LsKN1 bound to the *LsCUC3* promoter between the ‐2205 and ‐1790 bps (Figure 4c).

We carried out dual‐luciferase assay (LUC) to investigate the effects of LsKN1 on the expression of *LsCUC3*. The *LsKN1* gene bolstered the activity of *LsCUC3* promoter significantly (Figure 4d). Surprisingly, LsKN1 and LsKN1▽ showed similar binding ability to the promoter of *LsCUC3* (Figure S4a). The expression level of *LsCUC3* in the *LsKN1▽* genotypes with palmately lobed leaves was significantly higher than that in the *LsKN1* genotypes with pinnately lobed leaves from the F_3_ family. The above data were all testament to the LsKN1’s upregulation of *LsCUC3* (Figure 4e).

We hypothesized that the high expression of the *LsCUC3* gene triggered by

*LsKN1▽* contributed to the development of palmately lobed leaves. The overexpression of the *LsCUC3* gene in PINN led to palmately lobed leaves (Figure 4f; Figure 4g). On the contrary the knock‐out of the *LsCUC3* gene in PALM resulted in pinnately lobed leaves, in contrast to palmately lobed leaves in PALM (Figure 4h). Our results showed that *LsKN1* controlled palmately lobed leaves through *LsCUC3*, which is recessive epistatic to *LsKN1* for its effects on the development of palmately lobed leaves.

**Figure 5:**
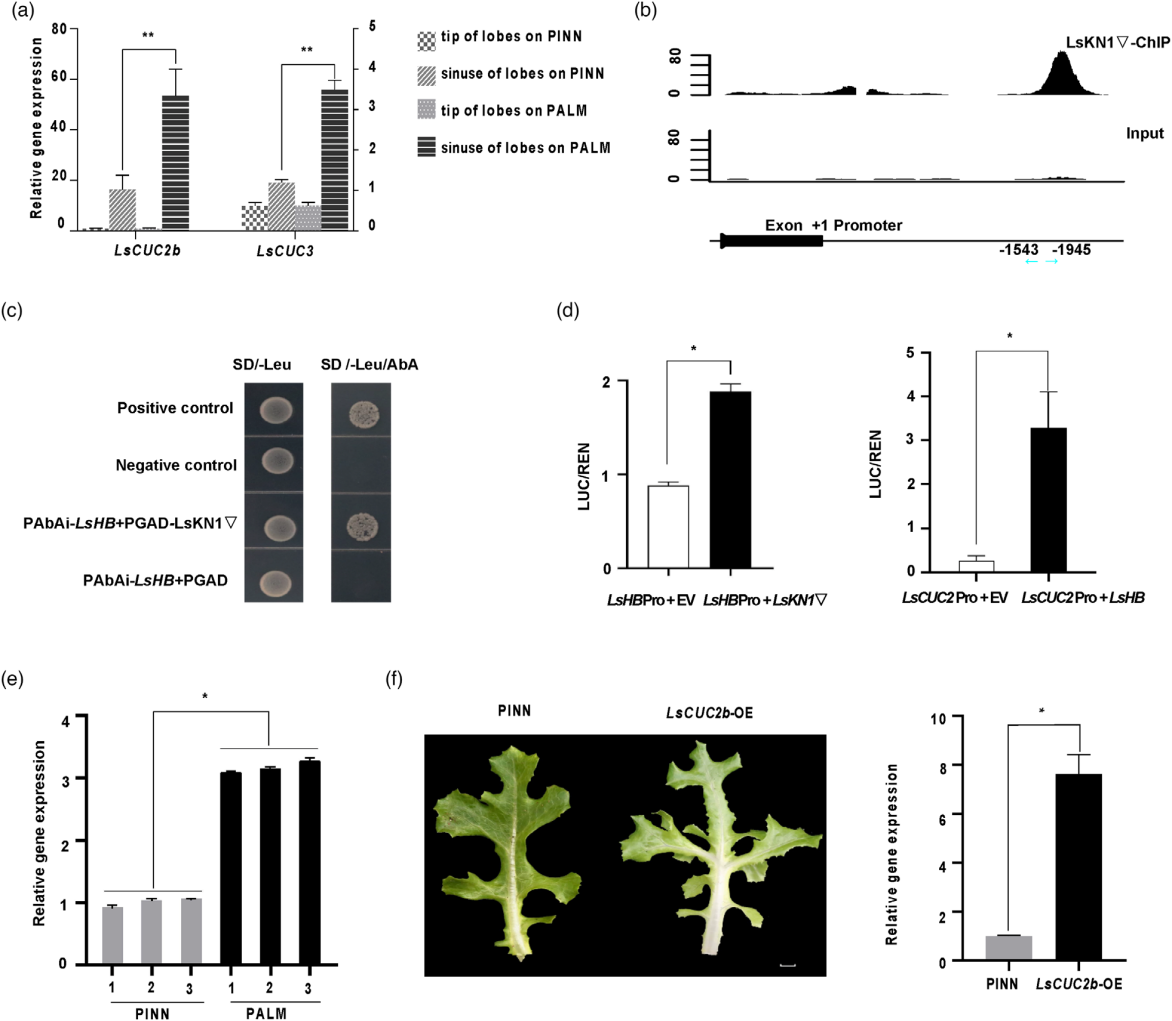
LsKN1 upregulates the expression of the *LsCUC2* gene through *LsHB*. (a) qRT‐PCR analysis of *LsCUC3* and *LsCUC2b* in PINN and PALM. Data represent mean ± SD (n = 3). ** denotes significance level of *P* < 0.01. The *LsCUC* genes are mainly expressed in the sinuses of lobes in PINN and PALM. (b) ChIP‐seq analysis of LsKN1 binding activity in the promoter region of *LsHB*. The y‐axis represents the number of reads in the ChIP‐seq. The x‐axis shows the position of the reads in the gene. (c) Y1H assay for LsKN1 and the promoter sequences of *LsHB*. Transformants were grown on the SD/‐Leu medium with 300 ng/mL AbA. See Figure 4b for details. The region of promoter is shown in (b). (d) Dual‐luciferase assay. The left panel shows the LUC activity when the *LUC* gene driven by the promoter of *LsHB* was co‐expressed with an empty vector (EV) or *LsKN1*▽. The right panel shows the LUC activity when the *LUC* gene driven by the promoter of *LsCUC2b* was co‐expressed with an empty vector (EV) or *LsHB*. Data represent mean ± SD (n = 3). * denotes significance level of *P* < 0.05. (e) qRT‐PCR analysis of the expression of the *LsHB* gene in *LsKN1* and *LsKN1*▽ genotypes in the F_3_ family. Data represent mean ± SD (n = 3). * denotes significance level of *P* < 0.05. (f) Overexpressing *LsCUC2b* increased the complexity of leaf lobes (left). qRT‐PCR analysis of the *LsCUC2b* gene in overexpression line (right). Bar = 2 cm. RNA was extracted from the sinuses of lobes. Data represent mean ± SD (n = 3). * denotes significance level of *P* < 0.05.

Like the *LsCUC3* gene, *LsCUC2b* was differentially expressed between palmately lobed leaves and pinnately lobed leaves (Figure 5a). However, ChIP‐seq results suggested that *LsCUC2b* was not a target of LsKN1. In Arabidopsis, *AtHB1*, an HD Zip I transcription factor, bound to the promoter region of *CUC2* and miR164, directly and indirectly upregulating the expression of *CUC2* (Miguel *et al*., 2020). Interestingly, ChIP‐seq data suggested that *LsHB* was a target of LsKN1 in lettuce, although *LsCUC2b* was not (Figure 5b). Y1H assay showed that LsKN1 bound to the promoter region of *LsHB* (Figure 5c). LUC results showed that the LUC activity driven by the promoter of *LsHB* considerably increased when the *LUC* reporter vector co‐expressed with the *LsKN1* gene. Similarly, the LUC activity driven by the promoter of *LsCUC2b* considerably increased when the LUC reporter vector co‐expressed with the *LsHB* gene (Figure 5d). qRT‐PCR showed that the expression of *LsHB* in *LsKN1▽* genotypes with palmately lobed leaves was significantly higher than that in *LsKN1* genotypes with pinnately lobed leaves from the F_3_ family (Figure 5e). We concluded that *LsKN1* indirectly upregulated the expression of *LsCUC2b* through *LsHB* (Figure S4b).

We then investigated whether the upregulated *LsCUC2b* gene contributed to the development of palmately lobed leaves. Overexpression of the *LsCUC2b* gene in PINN converted pinnately lobed leaves to palmately lobed leaves, similar to the phenotypic changes in the *LsCUC3* overexpression lines (Figure 5f). Therefore, the upregulated *LsKN1* gene contributes to the development of palmately lobed leaves through both *LsCUC2* and *LsCUC3* genes, which are mainly expressed in the sinuses of lobes (Figure 5a).

### LsKN1▽ promotes auxin biosynthesis to enhance palmately lobed leaves

**Figure 6:**
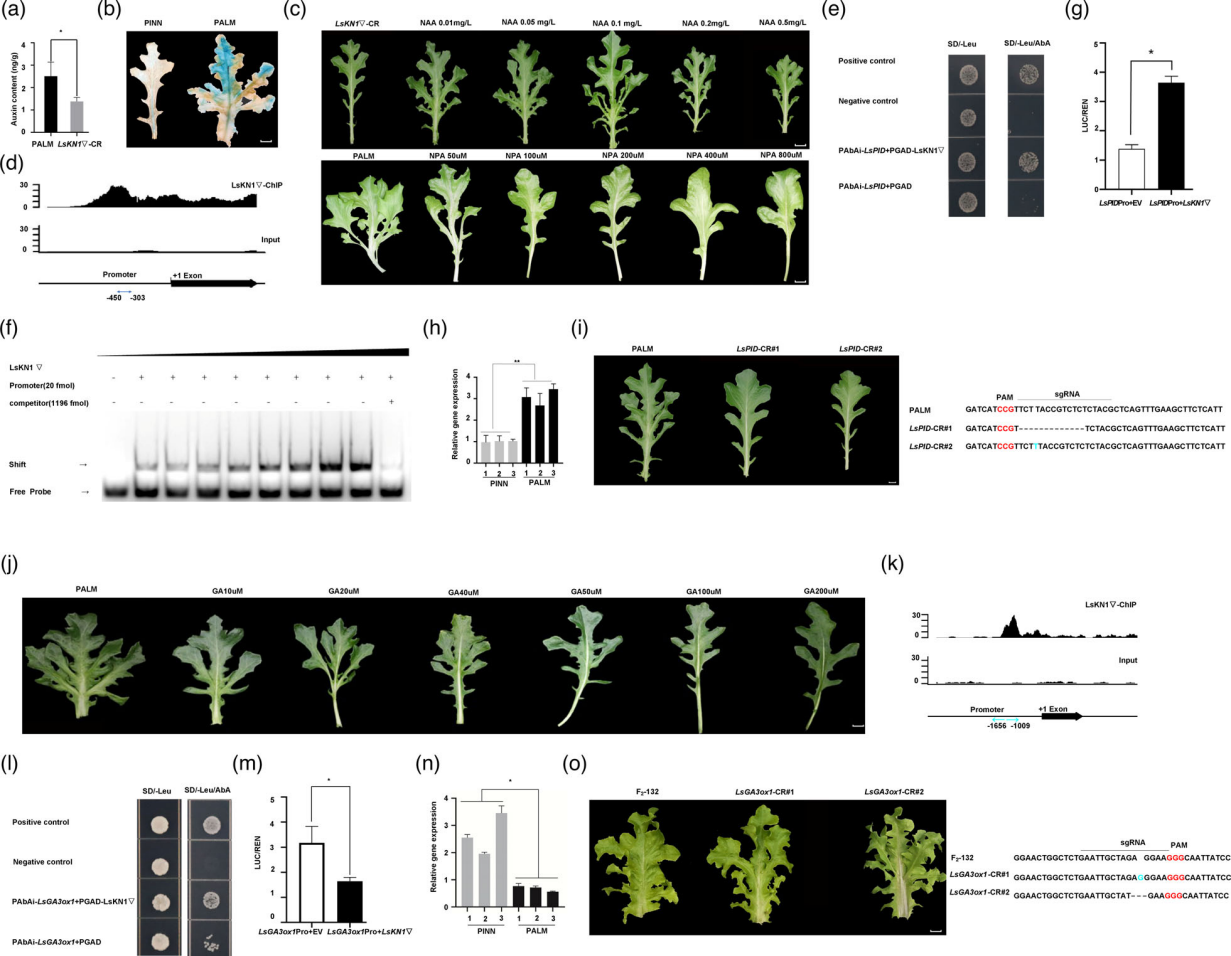
LsKN1 promotes palmately lobed leaves by promoting auxin biosynthesis and suppressing GA biosynthesis. (a) Auxin concentration in leaves of PALM and the *LsKN1*▽ knockout mutants. Data represent mean ± SD (n = 3). * denotes significance level of *P* < 0.05. (b) Auxin distribution indicated by *LsDR5::GUS*. Young leaves from one‐month‐old plants are shown. (c) Pinnately lobed leaves treated with auxin (top panel). Leaves from 2‐month‐old plants with pinnately lobed leaves were treated with different concentration of auxin. (n = 5). The bottom panel shows leaf changes after the palmately lobed leaves were treated with N‐1‐naphthylphthalamic acid (NPA), an auxin transport inhibitor. (n = 5). (d) ChIP‐seq results of LsKN1 on its potential target gene *LsPID*. The x‐axis shows the position of the reads in the gene. (e) Y1H assay for LsKN1 and the promoter sequences of *LsPID*. Transformants were grown on the SD/‐Leu medium with 300 ng/mL AbA. See Figure 4b for details. The region of promoter is shown in (d). (f) EMSA showing the binding of LsKN1 to the promoter of *LsPID*. (g) Dual‐luciferase assay. Compared with empty vector (EV), the *LUC* activity is lower when the *LUC* gene driven by the promoter of *LsPID* was co‐expressed with *LsKN1*▽. Data represent mean ± SD (n = 3). * denotes significance level of *P* < 0.05. (h) qRT‐PCR analysis of the *LsPID* gene in *LsKN1* and *LsKN1*▽ genotypes from the F_3_ family. Data represent mean ± SD (n = 3). ** denotes significance level of *P* < 0.01. (i) Knockout of *LsPID* changed palmately lobed leaves in PALM to pinnately lobed leaves (CR#1‐2) (left panel). Sequence modification in the *LsKN1* gene in the knockout plants (right panel). The sgRNA sequences are indicated with a horizontal line. Dash lines refer to deletion, and inserted nucleotides are in blue. PAM sequences are in red. (j) GA treatment on palmately lobed leaves. Leaves from 2‐month‐old plants with palmately lobed leaves were treated with different concentrations of GA. With the increase of GA concentration, palmately lobed leaves gradually converted to pinnately lobed leaves. (n = 5). (k) ChIP‐seq results of LsKN1 on the potential target *LsGA3ox1*. (l) Y1H assay for LsKN1 and the promoter sequences of *LsGA3ox1*. Transformants were grown on the SD/‐Leu medium with 400 ng/mL AbA. See Figure 4b for details. The region of promoter is shown in (k). (m) Dual‐luciferase assay. Compared with empty vector (EV), the LUC activity is lower when the LUC gene driven by the promoter of *LsGA3ox1* was co‐expressed with *LsKN1*▽. Data represent mean ± SD (n = 3). * denotes significance level of *P* < 0.05. (n) qRT‐PCR analysis of the *LsGA3ox1* gene in *LsKN1* and *LsKN1*▽ genotypes from the F_3_ family. Data represent mean ± SD (n = 3). * denotes significance level of *P* < 0.05. (o) Knockout of *LsGA3ox1* changed pinnately lobed leaves to palmately lobed leaves (CR#1‐2) (left panel). Sequence modification in the *LsGA3ox1* gene in the knockout mutants (right panel). The sgRNA sequences are indicated with a horizontal line. Dash lines refer to deletion, and inserted nucleotides are in blue. PAM sequences are in red. Bars = 2 cm.

Auxin modifications of leaf margins (Zhou *et al*., 2011). We found that the auxin content in the palmately lobed leaves of PALM is significantly higher than that in the pinnately lobed leaves of the *LsKN1* knockout mutants (Figure 6a). The high concentration of auxin in palmately lobed leaves was also demonstrated in the *LsDR5::GUS* transformants (Figure 6b). GUS activity was detected in all leaf veins in palmately lobed leaves, in contrast to limited to the main veins in pinnately lobed leaves. The activity of GUS at the margin of palmately lobed leaves was much higher than that of pinnately lobed leaves (Figure 6b).

To investigate whether auxin affects the complexity of leaf lobes in lettuce, we treated the leaves of PINN with Naphthaleneacetic acid (NAA). The pinnately lobed leaves changed to palmately lobed leaves after the treatment (Figure 6c). In contrast, the palmately lobed leaves in PALM changed to pinnately lobed leaves after it was treated with N‐1‐naphthylphthalamic acid (NPA), an auxin‐transport inhibitor (Figure 6c). We conclude that LsKN1 upregulates auxin biosynthesis, contributing to the development of palmately lobed leaves in lettuce.

The ChIP‐seq results suggested that LsKN1 binds to the promoter region of the *LsPID* gene (Figure 6d), which controls PIN polarity and mediates changes in auxin flow. Y1H (Figure 6e) assay and EMSAs (Figure 6f) showed that LsKN1 bound to the promoter region of *LsPID*. LUC assay showed that the LUC activity driven by the promoter of *LsPID* considerably increased when the LUC reporter vector co‐expressed with the *LsKN1* gene (Figure 6g). The LUC results indicated that LsKN1 upregulates the expression of *LsPID*. qRT‐PCR showed that the expression of *LsPID* in *LsKN1▽* genotypes with palmately lobed leaves was significantly higher than that in *LsKN1* genotypes with pinnately lobed leaves from the F_3_ family, consistent with the upregulation of *LsPID* by LsKN1 (Figure 6h).

We further knocked out the *LsPID* gene in the PALM line to confirm its function on lobe complexity. We constructed a recombinant CRISPR/Cas9 vector with sgRNAs specific to the coding region of the *LsPID* gene and transformed it into the PALM line. Two knockout lines were obtained, and both of them changed from palmately lobed leaves to pinnately lobed leaves (Figure 6i). Our knockout results further verified that the *LsPID* gene contributes to palmately lobed leaves in lettuce.

### LsKN1▽ suppresses GA biosynthesis to promote palmately lobed leaves

GA was shown to promote the elongation of plant leaves and inhibit leaf shape complexity (Hay *et al*., 2002; Smith *et al*., 1996). To investigate whether GA affects the complexity of leaf lobes in lettuce, we treated the leaves of PALM with GA. The results showed that the leaves changed to pinnately lobed with the increase of GA concentration in treatments (Figure 6j). The ChIP‐seq of LsKN1 suggested that LsKN1 bound to the promoter regions of *LsGA3ox1* and *LsGA20ox1*, two critical genes in the GA biosynthesis pathway (Figure 6k). However, the LUC assay could not confirm the regulatory role of LsKN1 on the expression of *LsGA20ox1* (Figure S4c). We hypothesized that the low expression of the *LsGA3ox1* gene triggered by LsKN1 contributed to the development of palmately lobed leaves. Y1H assay indicated that LsKN1 bound to the promoter region of *LsGA3ox1* (Figure 6l). LUC assay showed that the LUC activity driven by the promoter of *LsGA3ox1* considerably decreased when the *LUC* reporter vector co‐expressed with the *LsKN1* gene (Figure 6m). LUC assay showed that the proteins LsKN1 and LsKN1▽ had the same binding ability to the promoter of *LsGA3ox1* (Figure S4d). The LUC results indicated that *LsKN1* suppresses the expression of *LsGA3ox1*. qRT‐PCR showed that the expression of *LsGA3ox1* in *LsKN1▽* genotypes with palmately lobed leaves was significantly lower than that in *LsKN1* genotypes with pinnately lobed leaves from the F_3_ family (Figure 6n), consistent with above conclusion that LsKN1 suppresses the expression of *LsGA3ox1*.We knocked out the *LsGA3ox1* gene in a genotype with pinnately lobed leaves. The *Lsga3ox1* knockout mutant changed to palmately lobed leaves (Figure 6o). Our knockout results further verified that the downregulation of *LsGA3ox1* by LsKN1▽ contributes to palmately lobed leaves in lettuce.

### The effects of *LsKN1* on lobe complexity depend on *LsAS1* and *Lettuce Lobed Leaf* ( *LLL* ) genes

**Figure 7:**
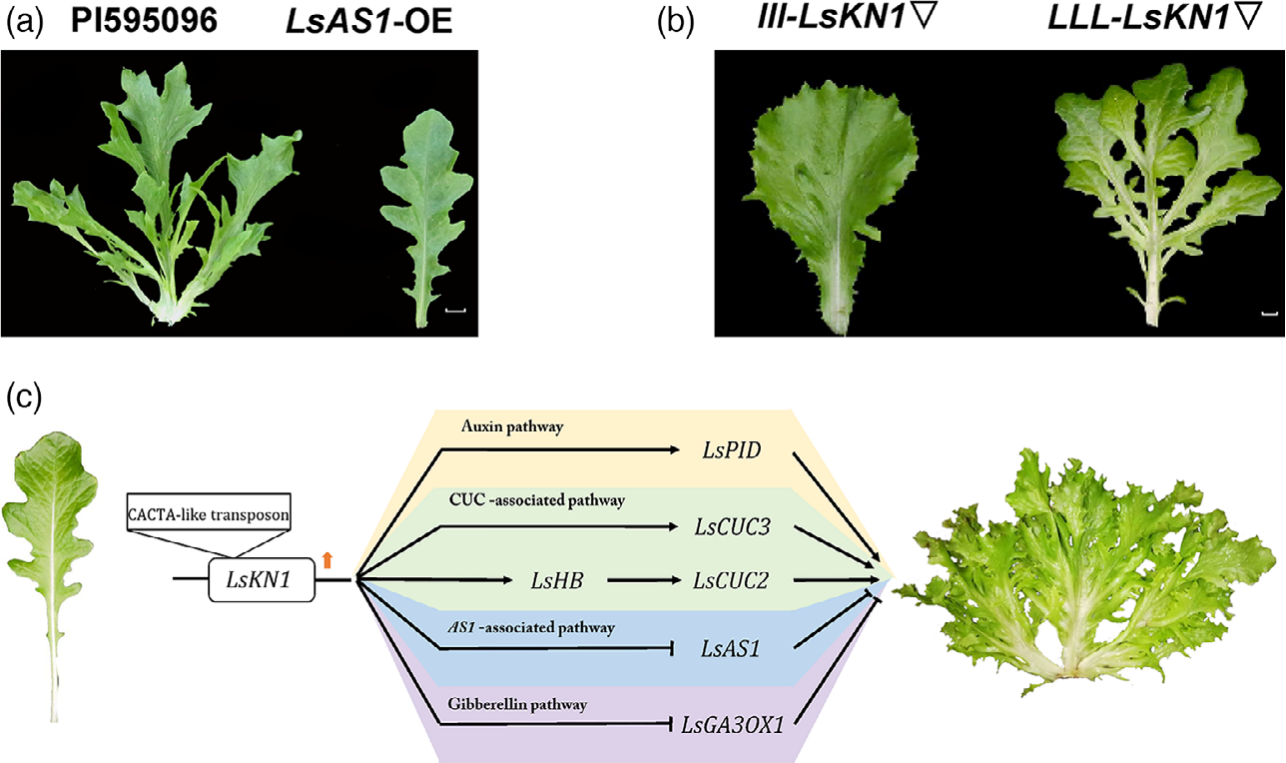
Effects of LsKN1▽ on palmately lobed leaves depend on *LsAS1* and *LLL*. (a) Overexpression of *LsAS1* in *LsKN1*▽ genotype changed palmately lobed leaves to pinnately lobed leaves. (b) The *Lettuce Lobed Leaves* (*LLL*) gene is recessive epistatic to LsKN1 in regulating palmately lobed leaves in lettuce. Bars = 2 cm. (c) The network of LsKN1 regulating palmately lobed leaves in lettuce.

Our previous study showed that LsKN1 promotes leafy heads through downregulating *LsAS1*, a critical gene controlling leaf dorsiventrality (Yu *et al*., 2020). *LsAS1* was the direct target of LsKN1, and it was downregulated in palmately lobed leaves compared to pinnately lobed leaves. To investigate whether *LsAS1* was required to develop palmately lobed leaves, we overexpressed *LsAS1* in cultivar PI595096, which had the *LsKN1▽* genotype and palmately lobed leaves. The overexpression line of *LsAS1* changed from palmately lobed leaves to pinnately lobed leaves, which was similar to the pinnately lobed leaves in *LsKN1* knockout mutants (Figure 7a).

It was shown recently that one single locus controls the polymorphism of lobed and non‐lobed leaves in Lactuca, but the causal gene (*Lettuce Lobed Leaves*, *LLL*) has not been identified (Wei *et al*., 2021). We crossed the FZ‐118 of palmately lobed leaves with a cultivar of non‐lobed leaves. As expected, the F_2_ population showed segregation of lobed and non‐lobed individuals. We used a highly linked marker to distinguish the *LLL* and *lll* haplotypes (Table S5). We compared the phenotypes of *PLL1/PLL2/PLL3* under the genetic background of *LLL* and *lll*. Interestingly, all *lll* homozygotes had non‐lobed leaves, while all *LLL* homozygotes and *LLL/lll* heterozygotes had palmately lobed leaves. Therefore, the *LLL* gene was recessive epistatic to *LsKN1* in regulation of palmately lobed leaves in lettuce (Figure 7b).

## Discussion

### The pleiotropism of *LsKN1▽* depends on genetic background

In our previous study, we crossed a romaine lettuce and a crisphead lettuce to construct a segregating population to investigate the genetics underlying leafy heads. We showed that the insertion of a CACTA‐like transposon upregulated the expression of the *LsKN1▽* gene to contribute to the development of leafy heads in lettuce (Yu *et al*., 2020). In the current study, we crossed a lettuce cultivar of palmately lobed leaves with a wild lettuce of pinnately lobed leaves to investigate the genetics underlying palmately lobed leaves. Surprisingly, the *LsKN1* gene is also responsible for the polymorphism of pinnately and palmately lobed leaves in lettuce, and the upregulated *LsKN1▽* allele controls palmately lobed leaves. The *LsKN1▽* allele, when introduced into stem lettuce, did not cause any noticeable phenotypic changes (Yu *et al*., 2020). Therefore, the *LsKN1▽* allele showed pleiotropic effects on the development of lettuce leaves, but the phenotype depended on genetic background. We predict that the multiple phenotypes derived from *LsKN1*▽, such as leafy heads and palmately lobed leaves, may occur in one plant if it has the corresponding genetic background, such as the *LLL* allele and other heading QTLs, respectively.

We showed that the *LLL* gene controlling lobed leaves was recessive epistatic to the *LsKN1▽* gene on palmately lobed leaves. Unfortunately, the *LLL* gene has not been cloned yet. With the identification of the *LLL* gene in the future, it will be interesting to investigate the molecular mechanism underlying the epistatic effects of *LLL* to *LsKN1*▽.

### LsKN1 controls palmately lobed leaves through multiple pathways

This study showed that LsKN1 controlled palmately lobed leaves through multiple pathways (Figure 7c). First, the auxin pathway plays important roles in the regulation of palmately lobed leaves triggered by *LsKN1*. ChIP‐seq results showed that the target genes of LsKN1 included *LsYUC4* (*LG197724*), *LsYUC2* (*LG8849068*), *LsYUC8* (*LG8736671*), *LsIAA3* (*LG8735159*), and *LsIAA9* (*LG3303130*), which are from the auxin biosynthesis and signaling pathways. The palmately lobed leaves of individuals with the *LsKN1▽* allele had significantly higher auxin concentration than the pinnately lobed leaves of individuals with the *LsKN1* allele. In Arabidopsis, the overexpression of the *YUC* genes led to the overproduction of auxin. *PIN1* and *YUC* genes synergistically control leaf development. The *yuc1 pin1‐5* double mutants have *pin*‐like leaves (Cheng *et al*., 2006; Wang *et al*., 2011). The mutation of a *YUC* homolog in Medicago suppresses lateral leaflet development (Zhao *et al*., 2020). The expression of *YUCs* in the leaves mediates the development of leaf margins and subsequently promotes blade outgrowth.

In contrast to auxin, GA reduces leaf complexity. The ChIP‐seq of LsKN1 suggested that LsKN1 bound to the promoter regions of *LsGA3ox1* and *LsGA20ox1*. Both *LsGA3ox1* and *LsGA20ox1* were differentially expressed between palmately lobed leaves and pinnately lobed leaves. Knockout of the *LsGA3ox1* gene in an individual with pinnately lobed leaves led to palmately lobed leaves. GA treatment of the Mouse‐ear mutant inhibited the super‐compound leaf phenotype of tomato (Hay *et al*., 2002). In Arabidopsis, KNOXI directly inhibits the activities of GA biosynthesis genes *GA3ox1* and *GA20ox1* to reduce the concentration of GA in SAM (Chen *et al*., 2004; Sakamoto *et al*., 2001).

The *ARP* (*AS1*/*RS2*/*PHAN*) gene family plays essential roles in maintaining stem cells and the initiation of lateral organs (Li *et al*., 2005; Timmermans *et al*., 1999; Tsiantis *et al*., 1999; Waites *et al*., 1998). Mutation of the *ARP* gene family affects not only cell properties but also leaf pattern formation. The *AS1* gene is expressed in leaf primordia, bract primordia, and flower organ primordia (Byrne *et al*., 2000; Tsiantis *et al*., 1999). In Arabidopsis, the down‐regulation of *STM* leads to the expression of the *AS1* gene at the leaf initiation site, while AS1 interacts with AS2 to inhibit the expression of other class I *KNOX* genes in the leaf primordium, resulting in leaf differentiation (Guo *et al*., 2008; Ori *et al*., 2000). Furthermore, AS1 and AS2 may form a complex to regulate the establishment of leaf polarity (Sun *et al*., 2002; Xu *et al*., 2003). The rosette leaves of the *as1* mutant are smaller and round, leaf margins are lobed, and leaf edges curl downward, resulting in asymmetric leaves (Byrne *et al*., 2000; Ori *et al*., 2000).

Our previous study has shown that LsKN1 promotes leafy heads by downregulating *LsAS1* (Yu *et al*., 2020). The current study revealed the involvement of the *LsKN1‐LsAS1* module in the development of palmately lobed leaves. Therefore, the *LsKN1▽* gene may regulate different traits (phenotypes) through similar pathways, and the ultimate phenotypes caused by the *LsKN1▽* gene are genetic‐background dependent.

### LsKN1 controls *LsCUC* homologs through different mechanisms

NAM/CUC is a vital transcription factor family regulating leaf lobes (Souer *et al*., 1996; Weir *et al*., 2004). CUC2 gene and the other two homologs CUC1 and CUC3 are necessary for organ boundary specification (Aida *et al*., 1997; Takada *et al*., 2001). It is believed that the leaf margin development of Arabidopsis is controlled through two steps. The pattern of serration is determined first, independent of CUC2 and miR164. The balance between CUC2 and miR164 then determines the degree of serration (Nikovics *et al*., 2006). During the development of leaf margin, CUC2 promotes the establishment of the *PIN1* convergence point, which produces the maximum auxin at the serrated end of leaf margin (Bilsborough *et al*., 2011; Cooke *et al*., 2002). Auxin maximally inhibits *CUC2* at the tip of the sawtooth and promotes tooth growth. While CUC2 is required early in serration formation, CUC3 acts later to maintain serration (Hasson *et al*., 2011).

The *LsKN1▽* genotypes had high expression of *LsCUC2b* and *LsCUC3*, both promoting leaf complexity. LsKN1 bound to the promoter region of the *LsCUC3* gene to upregulate its expression. In comparison, LsKN1 upregulated the expression of *LsHB*, which in turn upregulated the expression of *LsCUC2b*. In Arabidopsis, *AtHB* indirectly promotes the expression of *CUC2* by inhibiting miRNA164, and *AtHB* also directly activates the expression of *CUC2* to a certain extent (Miguel *et al*., 2020). Our study showed that *LsHB* in lettuce directly and significantly upregulated *LsCUC2b*.

In both *LsKN1* and *LsKN1▽* genotypes, the *LsCUC* homologs were only expressed in the sinuses of leaf lobes. The *CUC* homologs in Arabidopsis are also expressed in the sinuses, where growth and proliferation are reduced (Bilsborough *et al*., 2011; Kierzkowski *et al*., 2019; Nikovics *et al*., 2006). Interestingly, the high expression of *LsCUC* homologs in overexpression lines was also limited to the sinuses of leaf lobes but not in lobe tips. The uneven spatial distribution of *LsCUC* genes in leaves is most likely caused by miRNA164, which is present in lobe tips to silence the *CUC* genes (Hasson *et al*., 2011; Nikovics *et al*., 2006).

## Materials and methods

### Construction of F_2_ segregating population

An inbred line FZ118 with palmately lobed leaves was derived from the progeny of a looseleaf cultivar PI595096 and a crisphead cultivar PI64570A. The inbred line was crossed with *L. serriola* accession CGN04971, which has pinnately lobed leaves. PI595096 and PI64570A were ordered from the USDA GRIN (http://www.ars‐grin.gov/), while CGN04971 was ordered from CGN, Netherland (https://www.wur.nl/). The F_1_ hybrids were selfed to generate a segregating F_2_ population. The lettuce plants were grown on the campus of Huazhong Agricultural University in Wuhan, China.

### Genetic analysis of the loci controlling palmately lobed leaves

BSR genetic analysis followed the method described previously (Su *et al*., 2020). From the segregating population, 25 individuals with the most complex palmately (or pinnately) lobed leaves were chosen to construct a “palmately (or pinnately) lobe pool”. Equal amount of leaf tissues from the chosen individuals were mixed for each pool, and RNA was extracted using TRIzol reagent (Invitrogen). RNA was quantified and assessed using a Qubit Fluorimeter and a Nanodrop spectrophotometer (Novogene, Beijing, China). The non‐directional paired end RNA‐Seq libraries were constructed using the Illumina TruSeq RNA sample preparation kit, version 2, and sequenced using the Illumina HiSeq 2500 platform to obtain 125‐bp paired‐end reads. The raw date was filtered to remove low‐quality reads. Clean RNA‐seq data were mapped to the lettuce genome assembly v8 (Reyes‐Chin‐Wo *et al*., 2017), and SNPs were called. Allele (nucleotide) frequency for each SNP was calculated. The difference of allele frequencies between the two pools, ∆(SNP‐index), was plotted along the nine chromosomes of lettuce. A region with a high Δvalue was considered to potentially harbor a gene controlling the complexity of lobed leaves.

ANOVA model was used to quantify the effects of the candidate loci on leaf lobes using ANOVA function in R package ‘car’ (Fox *et al*., 2012). We used the number of lobes per leaf to represent leaf complexity. Leaf complexity for 237 individuals from the F_2_ population was determined using the number of lobes in the 8th leaf of the 45‐days‐old seedling. One CAPS marker was designed for each candidate locus and was genotyped for all individuals (Table S5). ANOVA was used to assess the association between candidate locus and leaf complexity, and then to calculate the variations of leaf complexity explained by the candidate loci.

### Quantitative reverse transcription PCR (qRT‐PCR)

Total RNA was extracted from the young leaves of 2.5‐month‐old plants using TriZol reagent (Invitrogen, Carlsbad, CA, USA), and treated with DNase I (ThermoFisher Scientific, https://www.thermofisher.com) to remove contaminated genomic DNA. First strand cDNAs were synthesized using a HiScript® II Q RT SuperMix for qPCR Reagent Kit (Vazyme Biotech, China). After confirming no genomic DNA contamination, qPCR was performed using the AceQ® qPCR SYBR Green Master Mix (Vazyme #Q111) in the LightCycler 480II System (Roche, Basel, Switzerland). qPCR was carried out using the 384‐well‐plate‐based real‐time PCR platform (Roche, Basel, Switzerland). *Actin* was used as a reference gene. Primers were designed using PREMIER 5.0 (Table S5).

Data represent mean ± SD (n = 3). The statistical significances for gene expression difference were determined using Student’s *t* test.

### Transformation and complementation tests

For complementation test, the full‐length of the *LsKN1* gene was PCR amplified from lettuce with palmately lobed leaves (FZ‐118) and purified using gel extraction kit (Omega bio‐tek, Norcross, USA), and then inserted into *Hin*dIII‐linearized binary vector pRI01C‐GFP using ClonExpress II One Step Cloning Kit (Vazyme, Nanjing, China). The resulting construct was transformed into *Agrobacterium* GV3101.

To overexpress the *LsKN1* gene, its full‐length cDNA sequence was inserted into the pHellsgate8 (Invitrogen, USA), pRI101‐GFP vector driven by the CaMV *35S* promoter. The construct was transformed into *Agrobacterium* strain GV3101 using the freeze‐thaw method. Transgenic plants were generated using cotyledon explants, cotyledon explants were infected with *Agrobacteria* (Michelmore *et al*., 1987), and were selected on MS medium supplemented with 60 mg/L kanamycin by the UC Davis Parsons Plant Transformation Facility (http://ucdptf.ucdavis.edu).

CRISPR‐Cas9 system was used to knock out target genes. gRNA sequences for CRISPR‐Cas9 were chosen using online program http://crispr.hzau.edu.cn/crispr2 (Liu *et al*., 2017). Four primers were designed for each gene, including BSF, F0, R0, and BsR, and the plasmids of pCBC‐DT1T2 vector as a template for amplification of the target fragment. pKSE‐401 vector was digested by *Bsa*I‐HF (New England Biolabs). Target fragment was homologous recombined with the linearized vector, then transformed into *E. coli* using heat shock method. The plasmid was extracted, and transformed into *Agrobacterium* using freeze‐thaw method.

### Dual‐luciferase assay

The dual‐luciferase reporter assay was performed as described previously (Hellens *et al*., 2005). The coding region of *LsKN1▽* was PCR amplified using the primers listed in Table S5. The PCR products were digested with *Spe*I and *Eco*RI and cloned into a pRTBD vector driven by a *35S* constitutive promoter to generate an effector plasmid. The primers listed in Table S5 was used to PCR amplify the natural *LsCUC3*, *LsHB*, *LsPID*, *LsCUC2b* promoter sequences, which was digested using *Hin*dIII and *Bam*HI, and inserted into a pGreen‐LUC vector to drive the firefly luciferase reporter gene as reporter plasmids. The plasmid containing the Renilla luciferase gene, driven by the *35S* promoter, was used as the control plasmid. The effector, reporter, and internal control plasmids were mixed at a ratio of 4:1. The mixtures were introduced into leaves of three‐week‐old *Nicotiana tabacum* using *Agrobacterium* infiltration. The activities of firefly and Renilla luciferase were quantified 2.5 d after infiltration with a Dual Luciferase Assay kit (Promega), and luminescence was recorded using a GloMax 96 Microplate Luminometer (Promega). The firefly luciferase activity (*LUC*) was normalized to the Renilla luciferase activity (*REN*). The assay was performed with three biological replicates, and the error bars represent the standard errors of the means from three independent experiments.

### Electrophoretic mobility shift assay (EMSA)

The EMSA assay was performed using the chemiluminescent EMSA kit according to a previously described method (Thermo, No 20148). The coding sequences of *LsKN1*▽were digested using *Eco*RI and cloned into a *E. coli* pMAL‐c2x (NEB) vector to generate the expression vector. MBP‐LsKN1▽ were purified by affinity purification with maltose. The expression of the MBP‐LsKN1▽ was expressed by induction with 1 mM IPTG at 16 °C for 8‐10 h in an orbital shaker. The bacteria were harvested by centrifugation and washed with prechilled PBS (137 mM NaCl, 2.7 mM KCl, 10 mM Na_2_HPO_4_, 2 mM KH_2_PO_4_, pH 7.4) and then resuspended in PBS. The *LsKN1▽* protein was purified from the crude extract using affinity chromatography with an amylose resin (NEB). To prepare the probe for EMSAs, fragments of *LsCUC3* promoter were PCR amplified and their 3’ ends were labeled with biotin according to the manufacturer’s recommendation (Thermo, No89818). The competitor was an unlabeled version of the same *LsCUC3* promoter fragment. The cold competitor was used to test whether LsKN1▽ specifically binds this probe. For the binding reaction, the LsKN1 protein, probes (20 fmol each), and competitor DNA (896 fmol) were incubated in the binding buffer (10 mM Tris‐HCl, pH 7.5, 50 mM KCl, 1 mM DTT, 2.5% Glycerol, 5 mM MgCl_2_, 0.05% NP‐40) with the presence of 3 μg/μL poly (dI•dC) at room temperature for 45 min. The complex was separated using 6% native SDS‐PAGE in 0.5X TBE buffer. The signal was detected using horseradish peroxidase (HRP) conjugated to streptavidin and an ECL substrate (Thermo, No 89880).

### Yeast‐one‐hybrid assay

Yeast‐one‐hybrid assay was performed according to the manufacturer’s instruction (Clontech). The coding region of *LsKN1▽* was digested with *Eco*RI and *Bam*HI and then cloned into a pGADT7 vector to generate a prey vector. After confirmation by DNA sequencing, the *E. coli* was incubated at 37 °C in dark for 24 h on LB media. Plasmid was extracted using Plasmid Mini Kit II D6945 (Omega). The natural *LsCUC3*, *LsHB*, *LsPID* promoter sequences were amplified using primers listed in Table S5, digested with *Xho*I and *Sac*I, and inserted into the bait vector (pAbAi). The bait vectors were confirmed through DNA sequencing. The *E. coli* was incubated at 37 °C in dark for 24 h on LB media. Plasmid was extracted using Plasmid Mini Kit II D6945 (Omega). The recombinant constructs were linearized using *Bst*BI and transformed into the Y1HGold yeast strain. After being cultured at 28 °C in dark for three days on Ura‐dropout media, the strains were screened using colony PCR. The second transformation recombinant constructs were the plasmid of prey vector and the bait‐reporter yeast strain. After confirmation by DNA sequencing, the plates were incubated for 3 days at 28 °C in dark on the Leu‐dropout media sift through aureobasidin A (AbA). We used transformants of p53‐AbAi and pGADT7‐p53 as a positive control, and transformants of p53‐AbAi and pGADT7 as a negative control.

## Accession numbers

All data supporting the results of this study can be found within and in the Supplementary table files. The raw data of ChIP‐seq, RNA‐seq of the BSR pools, and RNA‐seq of *LsKN1* knockout mutants and its recipients have been deposited in the National Center for Biotechnology Information (NCBI) under the BioProject ID PRJNA576072, PRJNA797355, and PRJNA797273, respectively. The sequences of *LsKN1*, *LsCUC3*, *LsHB*, *LsCUC2b*, *LsPID*, *LsGA3ox1* identified in this study are available in the NCBI GenBank (https://www.ncbi.nlm.nih.gov/genbank/) under the accession number LOC111890976, LOC111880143, LOC111896154, LOC111921537, LOC111880697, LOC111909350.

## Author contributions

M.W. performed the experiments; C.Y. helped on vector construction; D.L. and R.W.M. helped in genome sequences. W.Z. and X.W. did bioinformatic analysis. H.K. conceived and designed the experiment; M.W. wrote the manuscript with the help from H.K., J.C., X.W., R.W.M., and D.L.

## Competing interests

The authors declare no competing interests.

## Supporting information


**Figure S1** Alignment of LsKN1 and LsKN1▽ amino acid sequences.

**Figure S2** Venn diagram for DEGs for *LsKN1*. The blue circle is DEGs between *LsKN1*▽ and *LsKN1* plants that have non‐lobed leaves. The red circle is DEGs between *LsKN1*▽ and its knockout mutant, both with lobed leaves. The green circle represents potential targets of *LsKN1* according to ChIP‐seq results.

**Figure S3** Neighbor‐joining (NJ) phylogenetic tree for CUC homologs from lettuce and Arabidopsis. Amino acid sequences were used. Bar represents changes per site.

**Figure S4** Dual‐luciferase assay. (a) The left panel shows the diagram of reporter and effector vectors used in the dual luciferase assay. The LUC activity is similar when the *LUC* gene driven by the promoter of *LsCUC3* was co‐expressed with *LsKN1*▽ or *LsKN1*. Data represent mean ± SD (n = 3). * denotes significance level of *P* < 0.05. (b) The left panel shows the diagram of reporter and effector vectors used in the dual luciferase assay. The right panel shows that LsKN1▽ has no effects on the expression of *LsCUC2b*. Data represent mean ± SD (n = 3). *P* = 0.56 >0.05. (c) The left panel shows the diagram of reporter and effector vectors used in the dual luciferase assay. The right panel shows that LsKN1▽ has no effects on the expression of *LsGA20ox1*. Data represent mean ± SD (n = 3). *P* = 0.33 > 0.05. (d) The left panel shows the diagram of reporter and effector vectors used in the dual luciferase assay. LsKN1 and LsKN1▽ have similar effects on the expression of *LsGA3ox1*. Data represent mean ± SD (n = 3). * denotes significance level of *P* < 0.05.

**Table S1** Annotation of genes in the candidate region.
**Table S2** 389 DEGs between the *LsKN1*▽ knockout mutant with pinnately lobed leaves and its recipient with palmately lobed leaves.
**Table S3** Candidate targets of LsKN1▽ obtained by ChIP‐seq analysis.
**Table S4** 581 DEGs between *LsKN1*▽ mutant and its wild type.
**Table S5** Primers used in this study.
