# Supplementary figures and images for: The upregulated LsKN1 gene transforms pinnately to palmately lobed leaves through auxin, gibberellin, and leaf dorsiventrality pathways in lettuce
Source: Plant Biotechnol J. 2022 Jul 2;20(9):1756–69. doi: 10.1111/pbi.13861 (PMC9398307; doi:10.1111/pbi.13861)

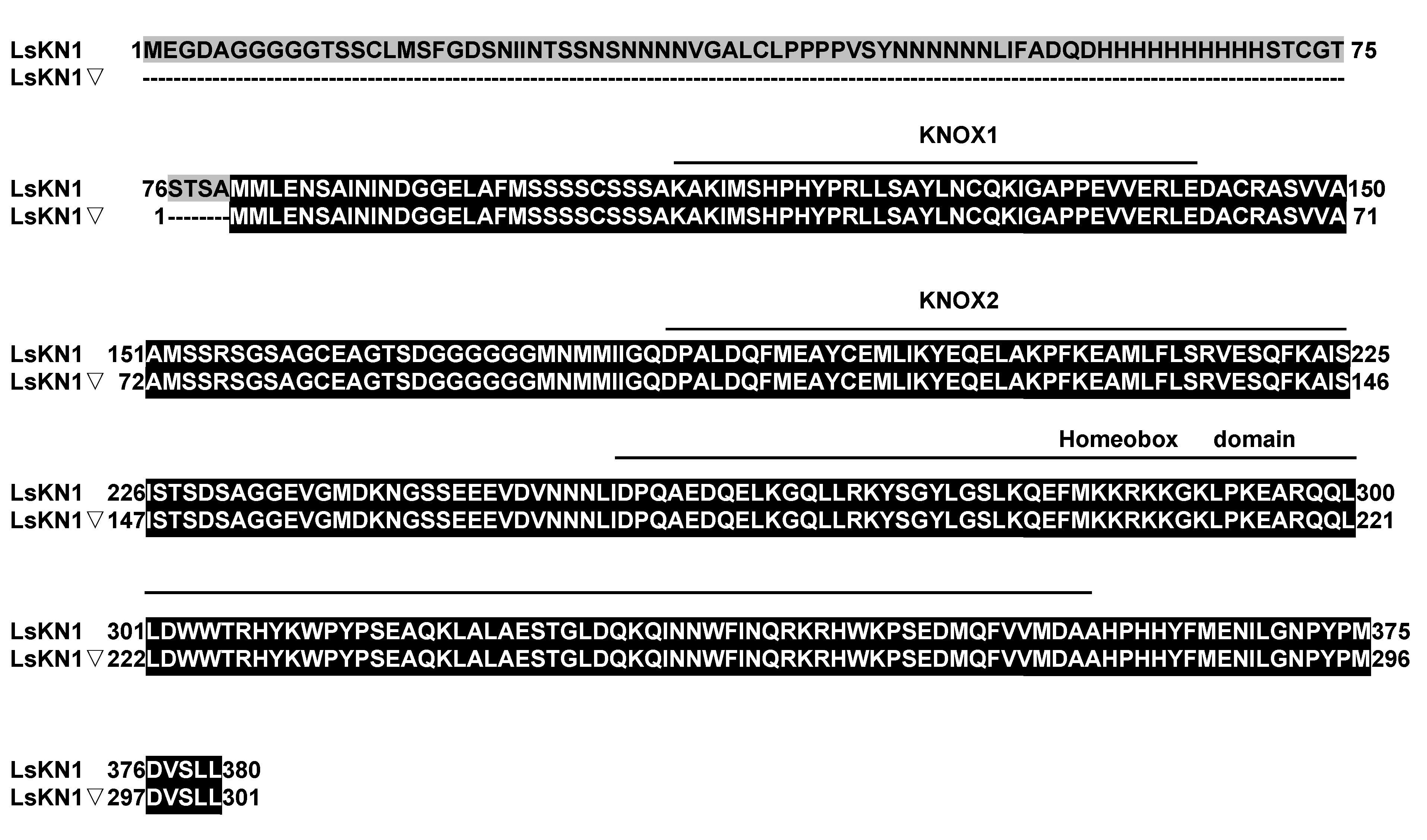

Supplement: Supplementary file 1 — Figure S1 Alignment of LsKN1 and LsKN1▽ amino acid sequences. [file PBI-20-1756-s001.tiff]

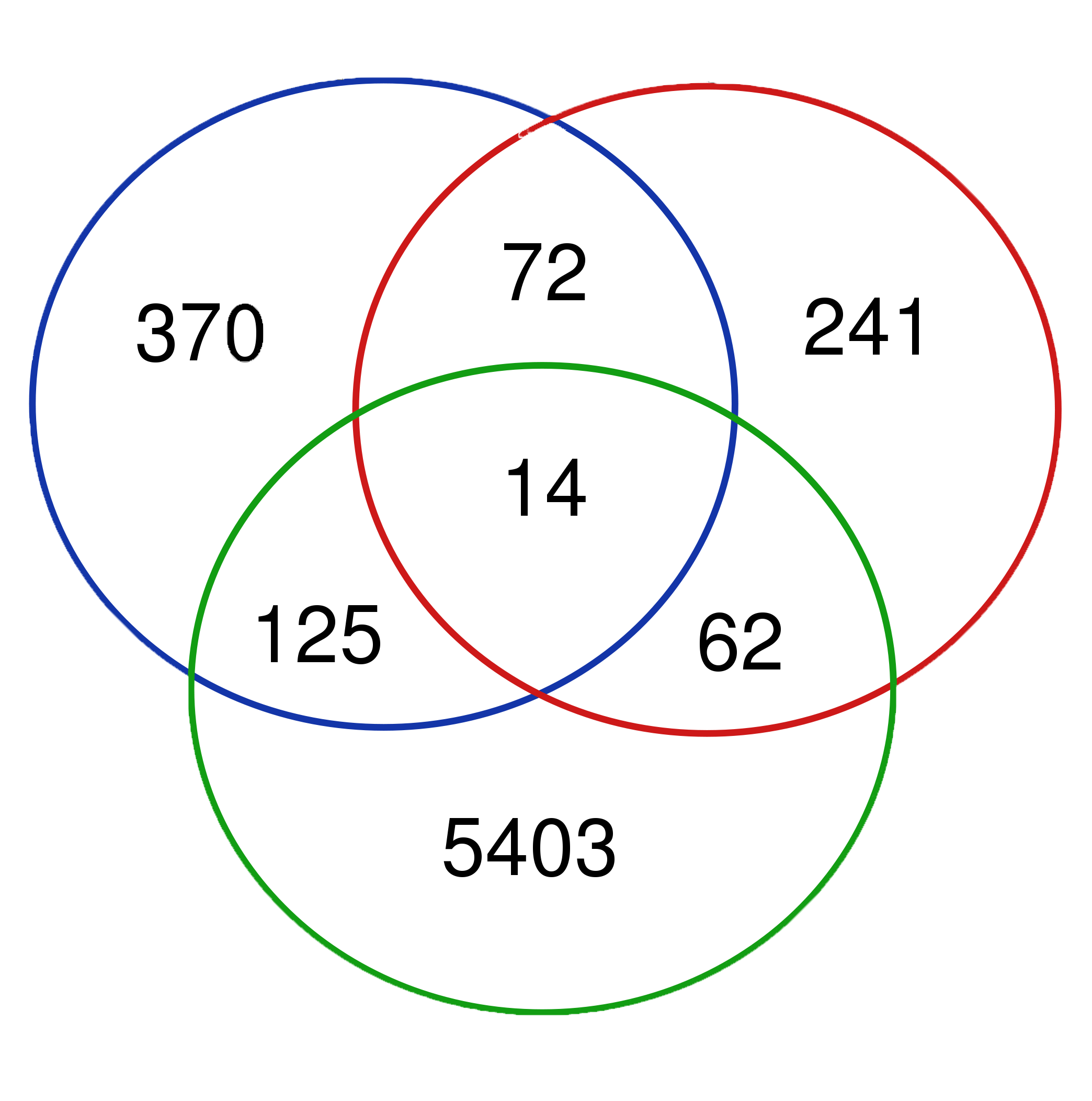

Supplement: Supplementary file 2 — Figure S2 Venn diagram for DEGs for LsKN1. The blue circle is DEGs between LsKN1▽ and LsKN1 plants that have non‐lobed leaves. The red circle is DEGs between LsKN1▽ and its knockout mutant, both with lobed leaves. The green circle represents potential targets of LsKN1 according to ChIP‐seq results. [file PBI-20-1756-s004.tiff]

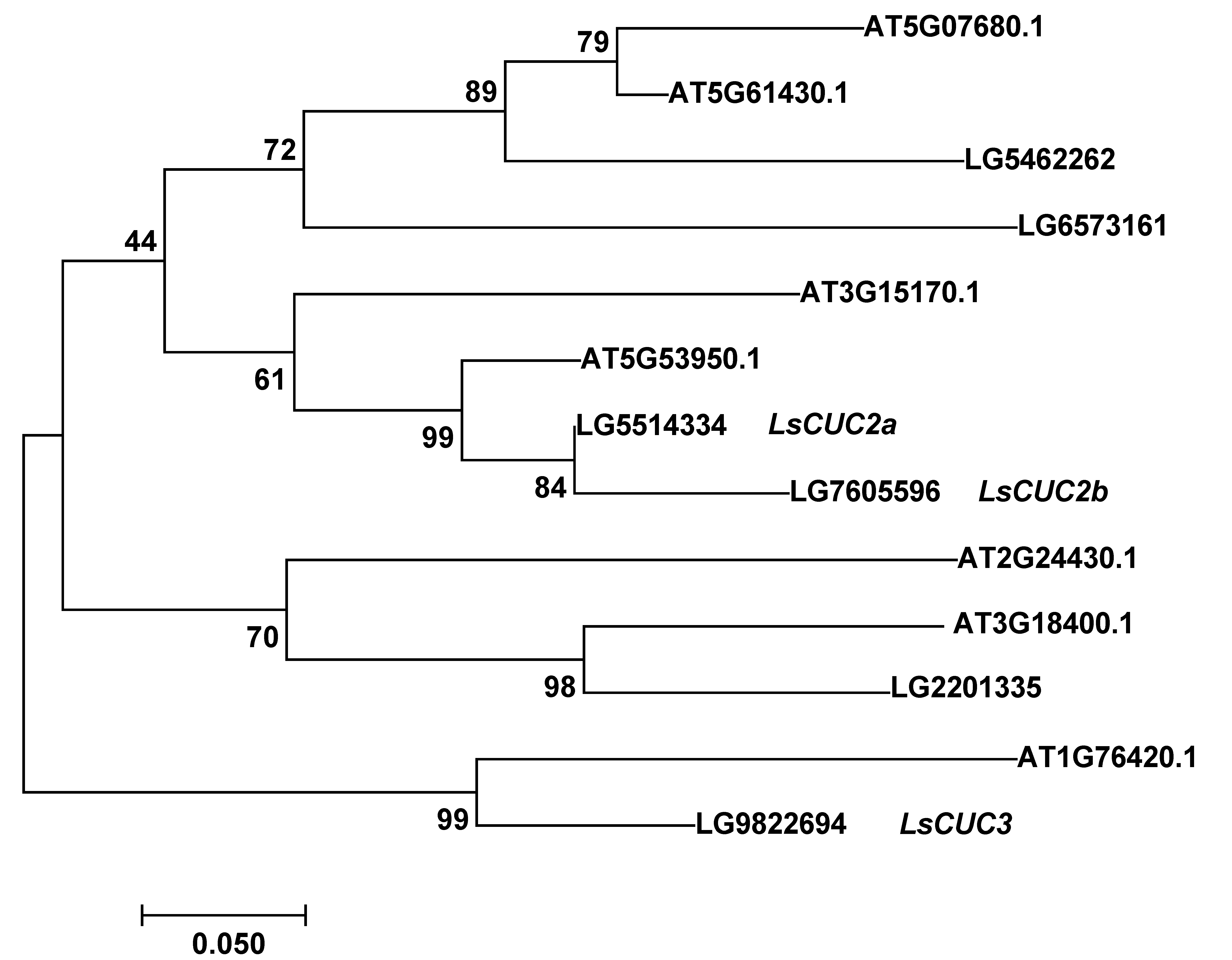

Supplement: Supplementary file 3 — Figure S3 Neighbor‐joining (NJ) phylogenetic tree for CUC homologs from lettuce and Arabidopsis. Amino acid sequences were used. Bar represents changes per site. [file PBI-20-1756-s002.tiff]

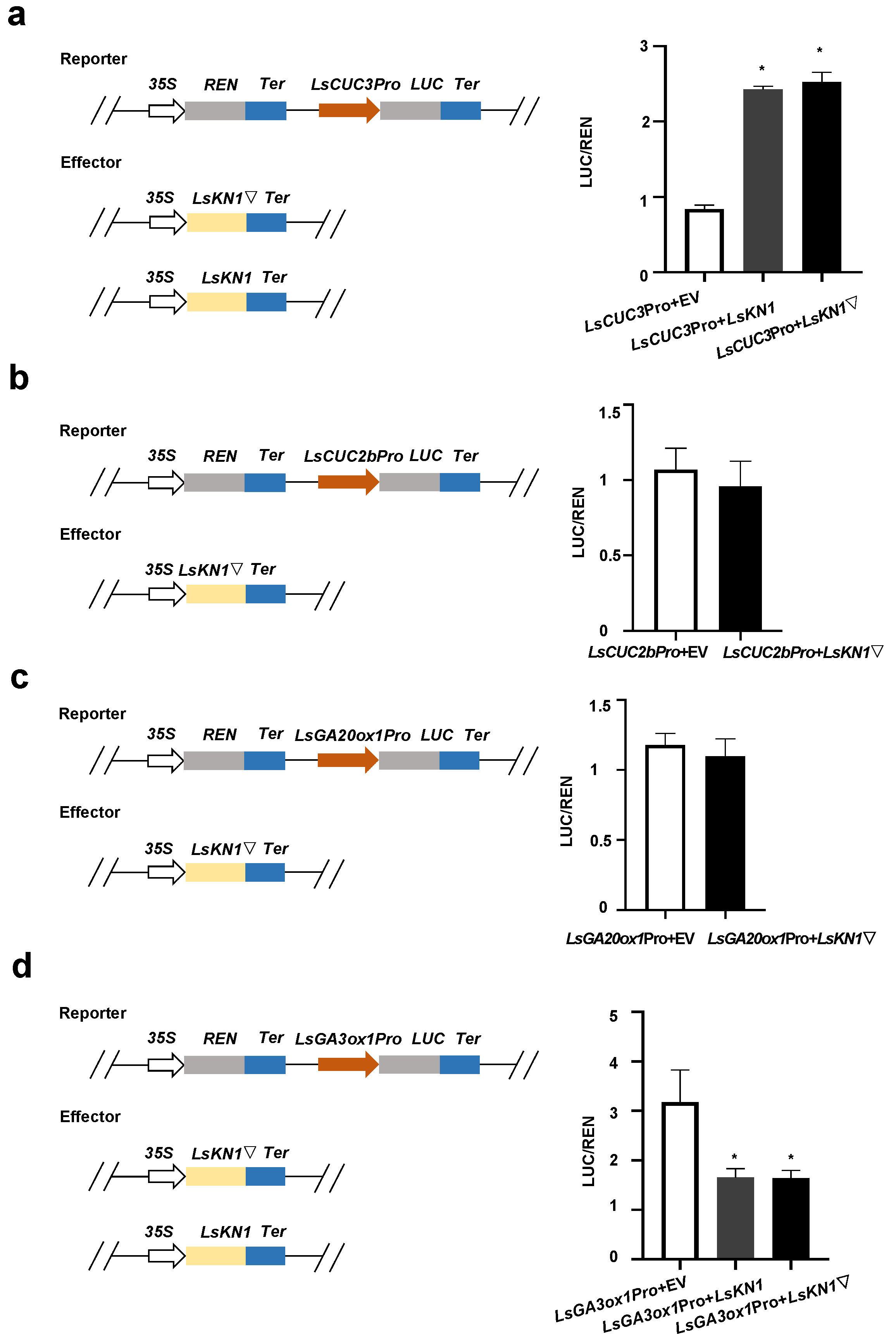

Supplement: Supplementary file 4 — Figure S4 Dual‐luciferase assay. (a) The left panel shows the diagram of reporter and effector vectors used in the dual luciferase assay. The LUC activity is similar when the LUC gene driven by the promoter of LsCUC3 was co‐expressed with LsKN1▽ or LsKN1. Data represent mean ± SD (n = 3). * denotes significance level of P < 0.05. (b) The left panel shows the diagram of reporter and effector vectors used in the dual luciferase assay. The right panel shows that LsKN1▽ has no effects on the expression of LsCUC2b. Data represent mean ± SD (n = 3). P = 0.56 >0.05. (c) The left panel shows the diagram of reporter and effector vectors used in the dual luciferase assay. The right panel shows that LsKN1▽ has no effects on the expression of LsGA20ox1. Data represent mean ± SD (n = 3). P = 0.33 > 0.05. (d) The left panel shows the diagram of reporter and effector vectors used in the dual luciferase assay. LsKN1 and LsKN1▽ have similar effects on the expression of LsGA3ox1. Data represent mean ± SD (n = 3). * denotes significance level of P < 0.05. [file PBI-20-1756-s005.tiff]
